# Bacteriophages with Potential to Inactivate *Aeromonas hydrophila* in Cockles: In Vitro and In Vivo Preliminary Studies

**DOI:** 10.3390/antibiotics10060710

**Published:** 2021-06-12

**Authors:** João Duarte, Carla Pereira, Pedro Costa, Adelaide Almeida

**Affiliations:** Department of Biology and CESAM, University of Aveiro, Campus Universitário de Santiago, 3810-193 Aveiro, Portugal; j.macedoduarte@ua.pt (J.D.); csgp@ua.pt (C.P.); pedrommrscosta@ua.pt (P.C.)

**Keywords:** *Aeromonas hydrophila*, bacteriophages, phage therapy, *Cerastoderma edule*, depuration, food safety

## Abstract

The recurrent emergence of infection outbreaks associated with shellfish consumption is of extreme importance for public health. The present study investigated the potential application of phages AH-1, AH-4, and AH-5 to inactivate *Aeromonas hydrophila*, a causative agent of infections in humans associated with bivalve shellfish consumption. The inactivation of *A. hydrophila* was assessed in vitro, using a liquid culture medium, and in vivo, using artificially contaminated cockles with *A. hydrophila* ATCC 7966. In the in vitro experiments, all phages were effective against *A. hydrophila*, but phage AH-1 (with a maximum reduction of 7.7 log colonies forming units CFU/mL) was more effective than phages AH-4 and AH-5 (with reductions of 4.9 and 4.5 log CFU/mL, respectively). The cocktails AH-1/AH-4, AH-1/AH-5, AH-4/AH-5, and AH-1/AH-4/AH-5 were slightly more effective than the single phage suspensions. The phages presented a low emergence rate of phage-resistant mutants. When artificially contaminated cockles were treated in static seawater with phage AH-1, around 44% of the added *A. hydrophila* (1.0 log CFU/g) was inactivated. The results of this study suggest that phage therapy can be an effective alternative to control human pathogenic bacteria during depuration.

## 1. Introduction

Bivalves are an essential part of the human diet, and their commercial value continues to increase worldwide [1]. In 2018, world production of aquaculture bivalves reached 17.7 million tonnes [2].

Infectious human diseases resulting from the consumption of bivalve shellfish are a threat to public health and in many countries represent a significant cost to society [3,4]. These health hazards are largely due to the filter-feeding nature of bivalves, as they concentrate and retain bacterial pathogens which are often derived from the contamination of their harvesting areas [4]. These pathogens can then be transmitted to consumers, thus posing a high risk to public health [3,5].

*Aeromonas hydrophila* is widely distributed in the aquatic environment, being a common contaminant of seafood in diverse geographic areas [6,7,8,9,10]. This bacterium causes a wide spectrum of human infections including gastroenteritis, wound infection, pneumonia, meningitis, endocarditis, and septicaemia (especially in immunocompromised hosts) [6,11,12].

To protect public health and provide safe products to consumers, the European Union Member States have applied the regulations published by the European Commission for the production and marketing of shellfish (RC 853/2004, 2073/2005, and 2285/2015). The ideal procedure to safely obtain bivalves would be their cultivation and harvesting in areas that are not subject to any kind of contamination. However, this is unfeasible from a productive point of view due to the scarcity of such pristine areas [5]. Depuration is a legal requirement in a large number of countries for the marketing of fresh bivalves in order to reduce the number of microorganisms to levels legally acceptable for human consumption and, consequently, to protect consumers’ health [3,13]. Briefly, depuration is a controlled process in which bivalves are reared in seawater treated with chlorine, ozone, iodine, or UV light for 24–48 h [13,14]. However, bacteria show different sensibilities to the depuration treatment. Some microorganisms are resistant to this process, persisting and multiplying in the shellfish tissue [15,16,17,18,19,20,21]. Additionally, the chemical treatments commonly used for seawater disinfection (e.g., chlorine or ozone) may be toxic for aquatic animals, unfavourable to their growth, and affect both the water-filtering activity of bivalve shellfish and the organoleptic quality of bivalves [14,22]. In order to reduce the concentration of potential human pathogens, the development and evaluation of new decontamination strategies with no adverse effects on bivalves is therefore essential. An efficient, environmentally friendly, and scientifically demonstrable solution to control bacterial contaminations in bivalve shellfish is phage therapy. Phages are viruses that only infect bacteria, being abundant in the environment and assuming a relevant role in bacterial population control in natural systems [23]. These viruses are target-specific and self-replicating rapid bactericides that do not affect animal cells [24]. Currently, the potential use of phage therapy in agriculture, veterinary biocontrol, food safety, and in the clinical treatment of human infections is being studied worldwide. Over the last few years, the use of phages on inactivate pathogenic bacteria in bivalves has gained momentum due to their inherent low toxicity [25]. Four studies have already reported the simultaneous use of depuration and phage biocontrol to eliminate pathogenic bacteria in bivalves [19,26,27,28]. For the first time, Rong et al. (2014) have evaluated the application of phages during the process of depuration on artificially contaminated oysters. Their results demonstrated that the application of phage VPp1 could reduce the population of *Vibrio parahaemolyticus* (with a maximum decrease of approximately 2.8 log CFU/g) in infected oysters [19]. Jun et al. (2014) have also reported the decrease of *V. parahaemolyticus* (by approximately 5.8 log CFU/g at an MOI of 10) after 72 h of phage pVp-1 application on artificially contaminated oysters in static systems [26]. Pereira and co-workers have revealed the viability of phage application to decrease bacterial population in both artificially and naturally contaminated bivalve shellfish during pilot-scale depuration, simulating the mechanical depuration strategies currently used [27,28]. These studies have shown that phage application during depuration procedures improves the microbiological safety of bivalves for human consumption by bolstering decontamination effectiveness, subsequently demonstrating that this technology can be carried out in the bivalve shellfish industry [27,28]. Additionally, this methodology cuts the time needed for depuration and, thus, diminishes its related expenses [27,28]. Be that as it may, these studies are limited to three bacterial strains (*V. parahaemolyticus*, *Escherichia coli*, and *Salmonella enterica* serovar Typhimurium), excluding some other important bacteria implicated in human infectious diseases transmitted by bivalve consumption such as *Aeromonas*, (namely, *A. hydrophila)* [3,29,30]. Therefore, the aim of this study was to evaluate the efficiency of three new phages (AH-1, AH-4, and AH-5) to control *A. hydrophila* in order to study their potential application during the depuration process. Several phages infecting *A. hydrophila,* isolated from different environments around the world, have already been characterized [29,31,32,33,34,35,36,37,38,39] and have been successfully used to treat infections [29,32,33,34,36,40,41,42,43]. The assays were performed in vitro and in vivo using artificially contaminated cockles with *A. hydrophila*.

## 2. Results

### 2.1. Phage Isolation and Enrichment

**Figure 1:**
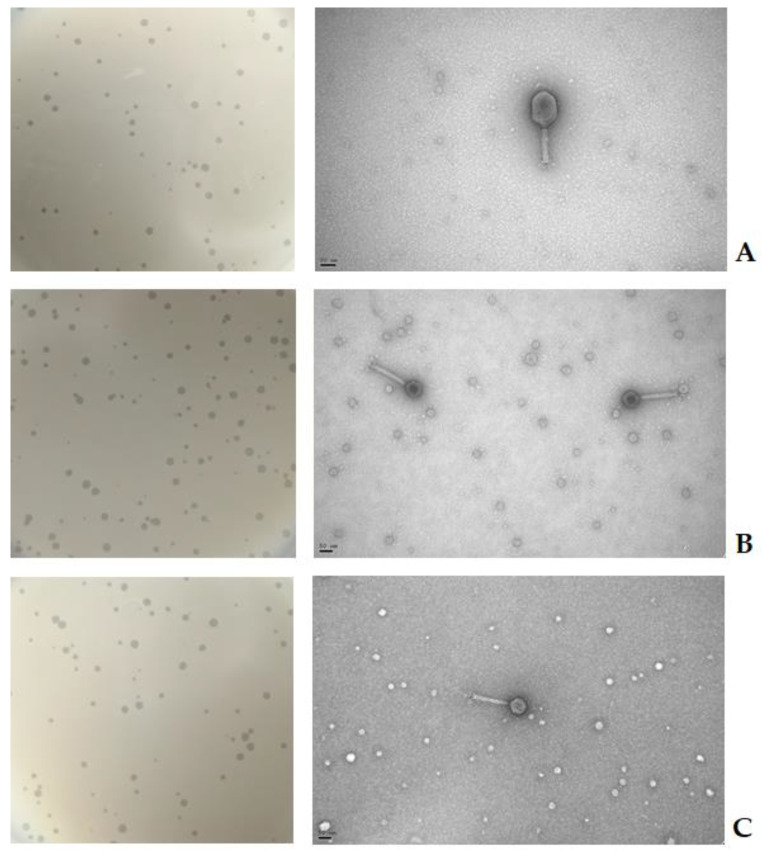
Phage plaque morphologies and electron micrographs of *A. hydrophila* phages: (**A**) Phage AH-1; (**B**) Phage AH-4; and (**C**) Phage AH-5. The bars represent 50 nm.

Phages AH-1, AH-4, and AH-5 formed clear plaques on the host strain with a diameter of 0.5–2 mm (Figure 1). High titre suspensions [10^9^ plaque-forming units (PFU/mL)] were produced for the three phages.

### 2.2. Virion Morphology

Based on the morphological analysis by TEM (Figure 1), all phages were identified as order Caudovirales and family *Myoviridae* of double-stranded DNA phages (Figure 1). Phage AH-1 has a rigid and contractile tail and an elongated icosahedral head of 75 ± 3 nm width and 104 ± 4 nm long. Phages AH-4 and AH-5 have an icosahedral head with 57 ± 2 nm width and a contractile tail ranging from 130 ± 10 nm.

### 2.3. Phage Host Range Determination and Efficiency of Plating (EOP) Analysis

Spot tests indicate that phages AH-1, AH-4, and AH-5, besides their host, could form completely cleared zones on 2, 3, and 1 of the 19 strains tested, respectively (Table 1). The phage AH-1 infected *S. typhimurium* ATCC 13311 and *A. hydrophila* 839, presenting an efficiency of 56.10 and 83.33%, respectively (Table 1). Phage AH-4 infected Bioluminescent *E. coli, S. typhimurium* ATCC 13311, and *A. hydrophila* 839 with an efficiency of 3.31, 10.17, and 91.14%, respectively (Table 1). Phage AH-5 infected only *A*. *hydrophila* 839 with 76.19% efficacy.

### 2.4. One-Step Growth Curve Analysis

**Figure 2:**
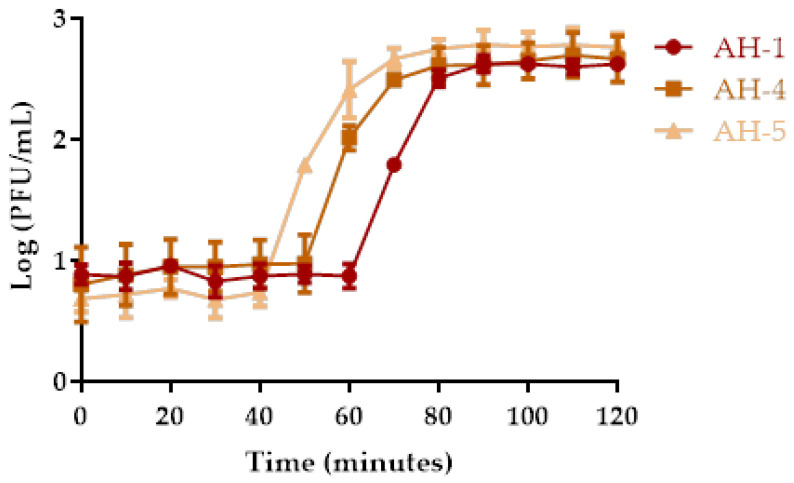
One-step growth curves of phages AH-1, AH-4, and AH-5 in the presence of *A. hydrophila* as the host. Values represent the mean of three independent experiments, and error bars represent the standard deviation.

Growth curves for phages AH-1, AH-4, and AH-5 were determined in TSB at 25 °C (Figure 2). From the triphasic curves obtained, phage AH-1 presented an eclipse time of 60 min, a latent period of 80 min, and a burst size of 39 ± 5 PFU/host cell. Phage AH-4 is characterized by an eclipse time of 50 min, a latent period of 70, and a burst size of 51 ± 9 PFU/host cell. Phage AH-5 is characterized by an eclipse period of 40 min, a latent period of 70 min, and a burst size of 112 ± 5 PFU/host cell.

### 2.5. Bacterial Killing Curves

#### 2.5.1. Bacterial Killing Curves Using Single Phage Suspensions

**Figure 3:**
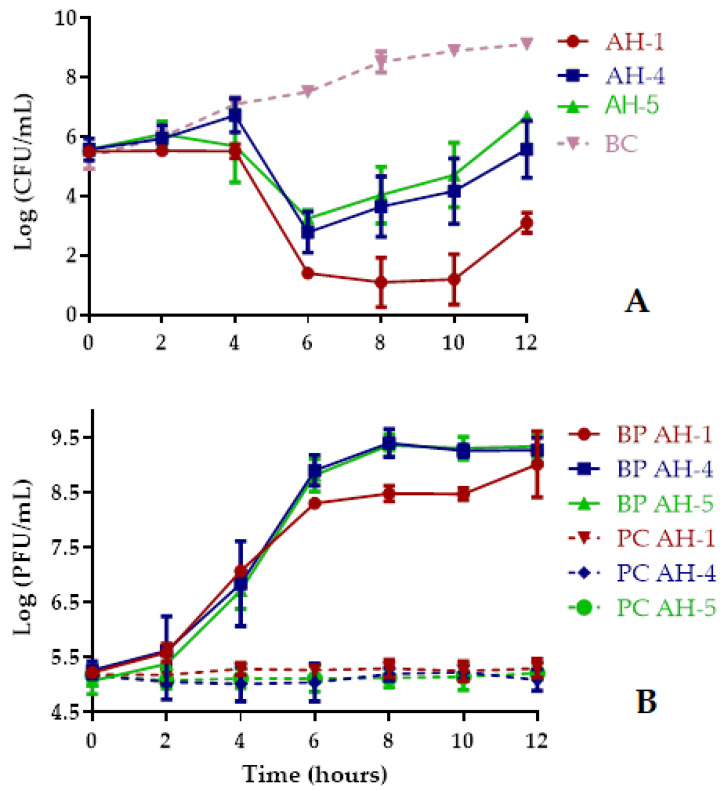
Inactivation of *A. hydrophila* by the three phages (AH-1, AH-4, and AH-5) during 12 h using an MOI of 1. (**A**) Bacterial concentration: BC, bacteria control; BP, bacteria plus phage. (**B**) Phage concentration: PC, phage control; BP, bacteria plus phage. Values represent the mean of the three experiments and error bars represent the standard deviation.

Bacterial density in the BC increased 3.8 log CFU/mL (ANOVA, *p* < 0.05, Figure 3A) during the 12 h of incubation. The maximum bacterial decrease with phages AH-1, AH-4, and AH-5 was, respectively, 7.7, 4.9, and 4.5 log CFU/mL (ANOVA, *p* < 0.05, Figure 3A), achieved after 8–10 h incubation when compared with those of the bacterial control (BC). However, after 6 h of incubation the inactivation rate was already 6.1, 4.8, and 4.3 log CFU/mL (ANOVA, *p* < 0.05, Figure 3A) for phages AH-1, AH-4, and AH-5, respectively. After 12 h of treatment, the rate of inactivation was still considerably high (ANOVA, *p* < 0.05, Figure 3A) for all phages (6.0, 3.5, and 2.4 log CFU/mL for phages AH-1, AH-4, and AH-5, respectively). The rate of bacterial inactivation with the phage AH-1 was, in general, significantly higher (ANOVA, *p* < 0.05, Figure 3A) than those obtained with phages AH-4 and AH-5. During the 12 h of incubation, the rate of bacterial inactivation with phages AH-4 and AH-5 was similar (ANOVA, *p* > 0.05).

No decrease in the phage survival was observed during the 12 h of the experiments for the phages alone, nor in the presence of the host (Figure 3B) in the different experiments. While the phage control (PC) remained constant throughout the experiment (ANOVA, *p* > 0.05), when phages AH-1, AH-4, and AH-5 were incubated in the presence of the host, a significant increase of 3.8, 4.0, and 4.3 log PFU/mL, respectively, was observed (ANOVA, *p* < 0.05, Figure 3B).

#### 2.5.2. Bacterial Killing Curves Using Phage Cocktails

**Figure 4:**
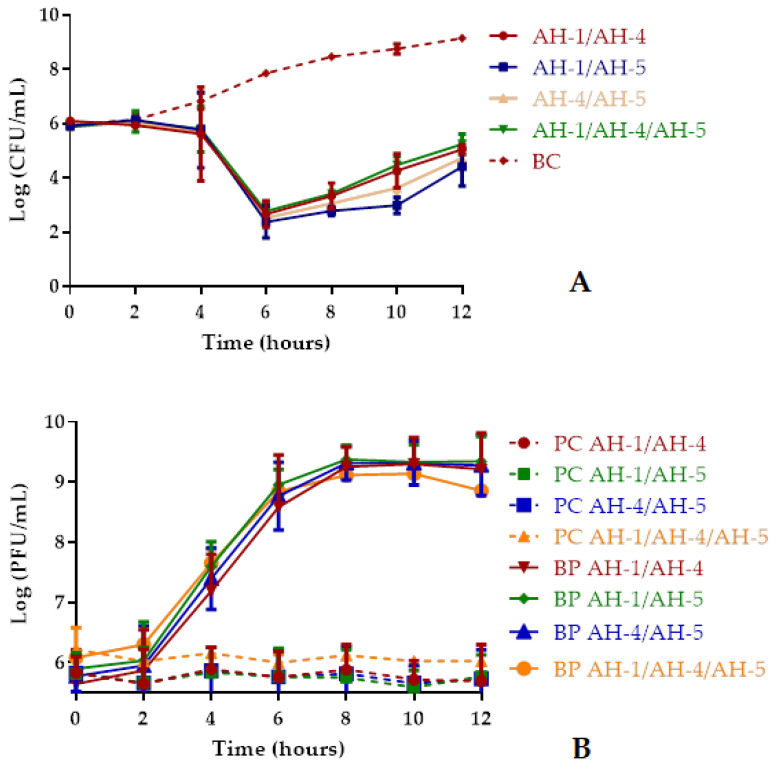
Inactivation of *A. hydrophila* by phage cocktails (AH-1/AH-4, AH-1/AH-5, AH-4/AH-5, and AH-1/AH-4/AH-5) at an MOI of 1 during 12 h. (**A**) Bacterial concentration: BC, bacteria control; BP, bacteria plus phage. (**B**) Phage concentration: PC, phage control; BP, bacteria plus phage. AH-1, phage AH-1; AH-4, phage AH-4; and AH-5, phage AH-5. Values represent the mean of the three experiments, and the error bars represent the standard deviation.

Bacterial density in the BC increased 3.2 log CFU/mL (ANOVA, *p* < 0.05, Figure 4A) during the 12 h of incubation. In general, the rates of inactivation were statistically similar (ANOVA, *p* > 0.05) for the four phage cocktails during the 12 h of incubation (Figure 4A).

The maximum inactivation of *A. hydrophila* with the phage cocktails AH-1/AH-4, AH-1/AH5, AH-4/AH-5, and AH-1/AH-4/AH-5 was 5.2, 5.8, 5.4, and 5.1 log CFU/mL achieved after 6, 10, 8, and 6 h of incubation, respectively, when compared with those of the bacterial control (BC) (Figure 4A). However, after 6 h the rate of inactivation was already considerably high (ANOVA, *p* < 0.05, Figure 4A) with the phage cocktails AH-1/AH-5, AH-4/AH-5, and AH-1/AH-4/AH-5 (5.5, 5.3, and 5.1 log CFU/mL, respectively). After 12 h of incubation, the rate of inactivation was still considerably high (4.1, 4.7,4.4, and 3.9 log CFU /mL for the phage cocktails AH-1/AH-4, AH-1/AH5, AH-4/AH-5, and AH-1/AH-4/AH-5, respectively, ANOVA, *p* < 0.05) (Figure 4A). The phage controls (PC) remained constant during the 12 h of the assay (ANOVA, *p* > 0.05, Figure 4B). In the case of the phage cocktails incubated in the presence of *A. hydrophila,* a significant increase (ANOVA, *p* < 0.05, Figure 4B) of 3.6, 3.5, 3.5, and 2.8 log PFU/mL was observed for AH-1/AH-4, AH-1/AH5, AH-4/AH-5, and AH-1/AH-4/AH-5, respectively, after 8 h of incubation.

### 2.6. Determination of the Rate of Emergence of Phage-Resistant Mutants

*A. hydrophila* showed different rates of phage-resistant mutants for single phage suspensions and phage cocktails (Table 2). The frequency of *A. hydrophila* mutants resistant to phage AH-1 (3.10 × 10^−3^) and AH-4 (1.14 × 10^−3^) was higher than the observed with phage AH-5 (5.02 × 10^−4^) and the phage cocktails (8.26 × 10^−4^, 6.40 × 10^−4^, 7.13 × 10^−4^, and 5.99 × 10^−4^ for the phage cocktails AH-1/AH-4, AH-1/AH-5, AH-4/AH-5, and AH-1/AH-4/AH-5, respectively).

### 2.7. Growth Curve of Phage-Resistant A. hydrophila Strains

**Figure 5:**
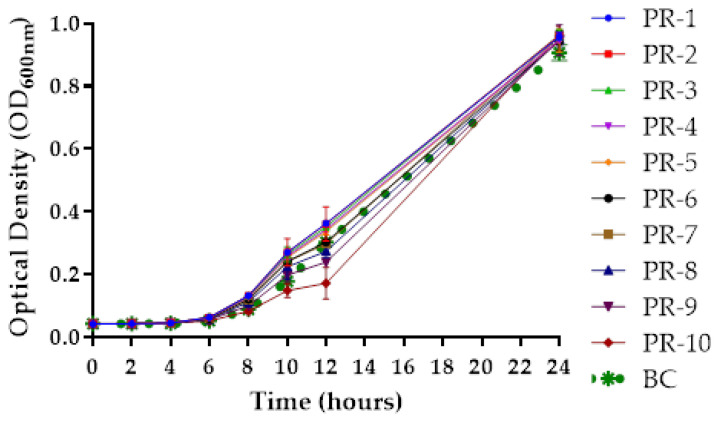
Growth curve of phage-resistant *A. hydrophila* strains during 24 h, with optical density (OD) readings at 600 nm. PR-1-PR10, phage resistant bacteria strains; BC, bacteria sensitive to phage AH-1. Values represent the mean of three three independent experiments and error bars represent the standard deviation.

The resistant mutants (PR1-PR10) retained resistance to AH-1 infection through 10 consecutive subcultures, and no morphological changes were observed in their colonies. The resistant mutants PR-8, PR-9, and PR-10 exhibited growth impairment after 12 h of incubation with the latter being the most affected, reaching less than 57% of the O.D.600 nm obtained by the parental strain (BC). However, after 24 h of incubation the growth of the resistant mutants (PR1-PR10) was similar to that obtained by the parental strain (BC) (Figure 5, ANOVA, *p* > 0.05). Phages AH-4 and AH-5 did not infect the phage AH-1-resistant mutants (PR1-PR10).

### 2.8. Bacterial Killing Curves at Different MOIs

**Figure 6:**
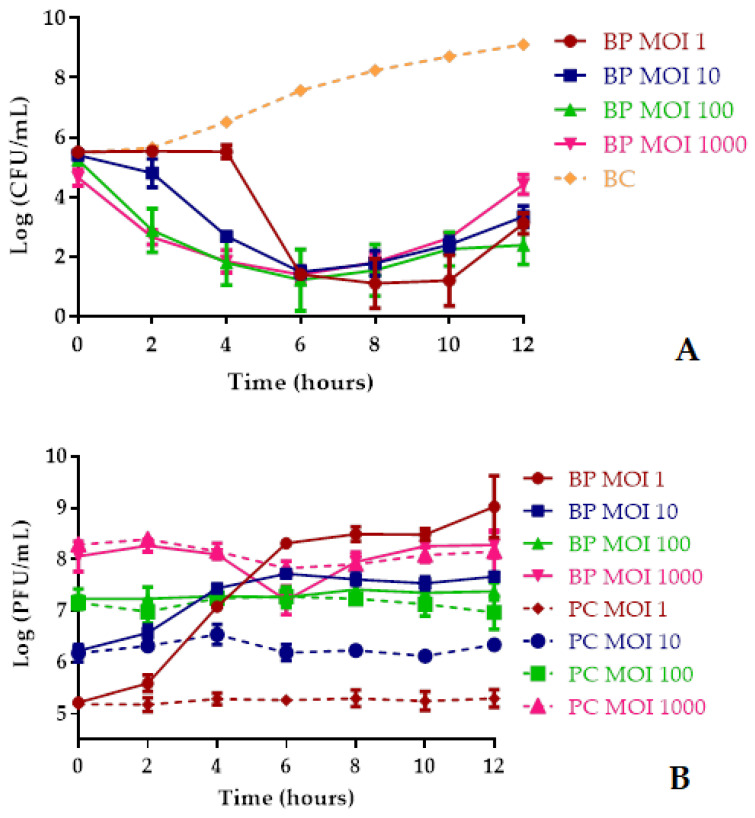
Inactivation of *A. hydrophila* by phage AH-1 at MOIs of 1, 10, 100, and 1000 during 12 h. (**A**) Bacterial concentration: BC, bacteria control; BP, bacteria plus phage. (**B**) Phage concentration: PC, phage control; BP, bacteria plus phage. Values represent the mean of the three independent experiments and error bars represent the standard deviation.

At MOIs of 1, 10, 100, and 1000, the maximum *A. hydrophila* inactivation with phage AH-1 was, respectively, 7.5 log CFU/mL after 10 h of incubation, and 6.5, 6.7, and 6.4 log CFU/mL, after 8 h of incubation when compared with those of the bacterial control. However, after 2 h the rate of inactivation was still considerably high (2.8 and 3.0 log CFU/mL; ANOVA, *p* < 0.05) for the MOI of 100 and 1000, respectively (Figure 6A). During the first 4 h of incubation, the increase in MOI significantly increased the inactivation factor (ANOVA, *p* < 0.05). However, after 4 h of incubation, there were no significant differences within the different MOIs (ANOVA, *p* > 0.05). After 6 and 8 h, the rate of inactivation was similar for all tested MOIs (Figure 6A). At the end of the experiment, the rate of bacterial inactivation with the MOI of 1 and 100 (reduction of 6.0 and 6.7 log CFU/mL, respectively) was significantly higher (ANOVA, *p* < 0.05) than the one obtained with the MOI of 10 and 1000 (5.8 and 4.7 log CFU/mL, respectively).

The bacterial density in the BC increased 3.8 log CFU/mL (ANOVA, *p* < 0.05) during the 12 h of incubation (Figure 6A). The phage concentration in the controls (PC) remained constant during the 12 h timeframe of the experiments (ANOVA, *p* > 0.05, Figure 6B) and when phage AH-1 was incubated in the presence of its host, a significant increase (ANOVA, *p* < 0.05) in the phage particle concentration (3.8 and 1.5 log PFU/mL after 12 and 6 h of incubation) was observed for the MOI of 1 and 10, respectively (Figure 6B). No significant difference was observed when phage AH-1 was incubated in the presence of the *A. hydrophila* (BP) at an MOI of 100 and 1000 when compared with the phage control (ANOVA, *p* > 0.05, Figure 6B).

### 2.9. Phage Application during Cerastoderma edule Depuration in a Static System

**Figure 7:**
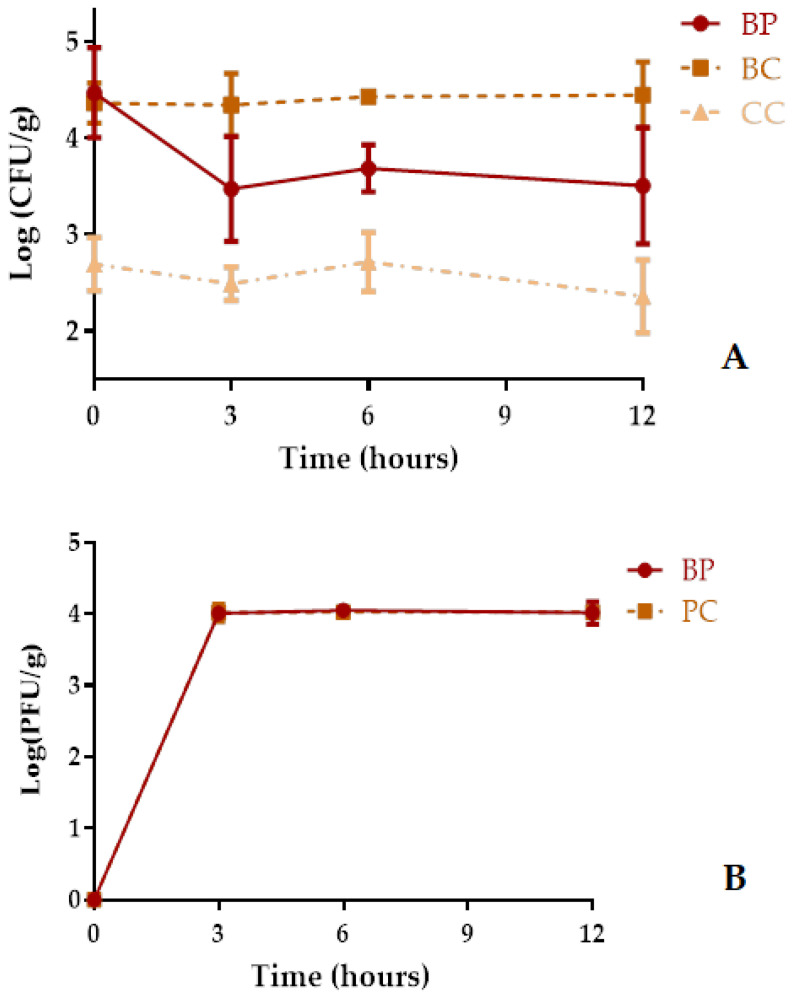
Inactivation of *A. hydrophila* in artificially contaminated cockles using phage AH-1 at a MOI of 1 during 12 h. (**A**) Bacterial concentration: CC, uninfected group (control cockles); BC, bacteria control; BP, bacteria plus phage. (**B**) Phage concentration: PC, phage control; BP, bacteria plus phage. Values represent the mean of the three experiments and error bars represent the standard deviation.

The total concentration of cultivatable bacteria present in the cockles at the start of the assay was 2.7 log CFU/g (Figure 7A) and no *Aeromonas* species were detected. The bacterial density in the bacterial control in the cockles at the start of the assay was 4.4 log CFU/g. The bacterial density in the bacterial control (BC) and animal control (CC) remained constant (ANOVA, *p* > 0.05, Figure 7A) during the 12 h of treatment.

The maximum rate of cultivable bacteria inactivation in cockles treated with phage AH-1, relatively to the bacterial control, was 1.0 log CFU/g (ANOVA, *p* < 0.05, Figure 7A) achieved after 12 h of treatment. However, after 3 h, the rate of inactivation was already considerably high (0.9 log CFU/mL; ANOVA, *p* < 0.05).

The abundance of phage AH-1 in cockles in the absence (PC) and presence of the host *A. hydrophila* (BP, Figure 7B) increased during the first 3 h and then remained constant until the end of the treatment.

## 3. Discussion

The elimination of *A. hydrophila* in bivalve molluscs is very important for public health. However, the treatments currently available for bivalves’ decontamination are not fully efficient nor environmentally safe. While some studies have demonstrated that phages can be used to successfully control pathogenic bacteria associated with shellfish consumption [19,26,28,44,45], no effort has been made to evaluate their effectiveness to control *A. hydrophila* in bivalves molluscs. In this study, the results showed that the application of phages could reduce the population of *A. hydrophila.*

Phage specificity is very important in phage therapy, and it was observed for the phage AH-5 tested in this study. However, phages AH-1 and AH-4, besides their host *A. hydrophila* ATCC 7966, can also infect other *A. hydrophila* strains and other bacterial species frequently associated with infectious outbreaks through bivalve shellfish consumption. Phage AH-1 infected *A. hydrophila* 839 and *S. typhimurium* ATCC 13311, and phage AH-4 infect *A. hydrophila* 839, an *E. coli* strain (bioluminescent *E. coli*) and a *Salmonella* strain (*S. typhimurium* ATCC 13311) which belong to different bacterial genera. Similar results were already observed in other studies [44,45,46]. These results suggest that phages AH-1 and AH-4 can be used not only to control *A. hydrophila* but also other human pathogenic bacteria associated with shellfish consumption, namely *E. coli* and *S. typhimurium*. According to Mirzaei and Nilsson (2015), a collection of phages with wide host ranges would facilitate the creation of phage libraries and reduce study costs [47]. The effectiveness of phages AH-1 and AH-4 was also tested against other *Aeromonas* species and other bacterial genera, but none of these bacteria was infected by the two phages (Table 1). The majority of marine phages are highly host-specific [48,49,50] and 73% of them lyse only the original host bacterium [50], as the phage AH-5. El-araby et al. (2016) demonstrated that two *A. hydrophila* phages infected only one *A. salmonicida* of the tested six strains and did not infect any isolates of non-*Aeromonas* bacteria tested [36]. In another study, Akmal et al. (2020) observed that the phage Akh-2 lysed only four *A. hydrophila* strains (four of the seven strains tested) and could not infect the other 23 strains tested [29]. In the future, new phages need to be isolated and tested together with phages AH-1, AH-4, and AH-5 to produce a cocktail with a broader spectrum of activity against *A. hydrophila* and even to control other pathogenic bacteria associated with shellfish consumption.

Some studies have demonstrated that phages with high burst sizes and short lytic cycles increase the efficiency of phage therapy [44,51,52]. However, high burst sizes are usually followed by a more extensive latency period [53]. Nevertheless, the true correlation between these properties and therapy success is not yet fully understood [54,55]. Although the burst size was higher for phage AH-5 (112 ± 5 PFU/host cell) than for phages AH-1 (39 ± 5 PFU/host cell) and AH-4 (51 ± 9 PFU/host cell) (Figure 2), the in vitro phage inactivation was higher with phage AH-1 (maximum inactivation of 7.7, 4.9 and 4.5 log CFU/mL for phages AH-1, AH-4, and AH-5, respectively, Figure 3A), suggesting that other factors regulate the phage–bacteria interaction. 

*A. hydrophila* was effectively inactivated by the three phages AH-1, AH-4, and AH-5 (Figure 3A), but the phage AH-1 was the most effective (with a maximum of inactivation of 7.7 against 4.9 and 4.5 log CFU/mL for AH-4 and AH-5 after 8–10 h of incubation; most of the inactivated bacteria did not regrow after treatment, i.e., when phage AH-1 was used). All phage cocktails tested (two or all three phages mixed) to inactivate *A. hydrophila* were more efficient to control the bacterial growth than phages AH-4 and AH-5 alone. These results are in accordance with other studies [28,51,56] that achieved a higher bacterial decrease by using phage cocktails, than that obtained with single phage suspensions. However, phage AH-1 (with a maximum reduction of 7.7 log CFU/mL (Figure 4A)) was more efficient than the other phages alone (with maximum reduction of 4.5–4.9 log CFU/mL (Figure 4A)) and phage cocktails (with a maximum reduction of 5.1–5.8 log CFU/mL (Figure 4A)). When the most efficient phage (AH-1) was used in the cocktails, the efficiency of the phage cocktails was not better than with phage AH-1 alone. This can be explained by the fact that phage AH-1 may target the same bacterial receptor as phages AH-4 and AH-5, expaining why phages AH-4 and AH-5 did not infect phage AH-1-resistant mutants (PR1-PR10). As phage cocktails, besides being used to increase bacterial inactivation and to increase the host range, are also used to delay the development of phage-resistant mutants, the type of bacterial receptor that each phage uses to infect its host should be considered when selecting the phages to be included in a specific cocktail. Filippov et al. (2011) showed that the use of phage cocktails can overcome the problem of bacterial resistance, but that is only the case if the phages exploit different receptors. Further studies, including the identification of the bacterial receptors used by the tested phages to infect *A. hydrophila,* are necessary to confirm this hypothesis [57].

When phage AH-1 was used, in general, the bacterial regrowth was lower than that observed when the other phages were tested alone or in phage cocktails. However, the use of phage AH-1, as well as the use of other single-phage suspensions and phage cocktails, did not prevent the occurrence of phage-resistant mutants (Table 2). Nonetheless, the frequency of phage-resistant mutations was low (10^−3^–10^−4^ CFU/mL) and was slightly lower for the phage cocktails than for the single phage suspensions (Table 2), as was observed in other studies [56,58,59]. With such a small mutation frequency, phage resistance should not hinder the use of phages as biocontrol agents against pathogenic bacteria, as has been stated before by other authors [60,61]. The diversity between different phage-resistant mutants can be studied through their growth curves. In this study, the resistant mutants PR-8, PR-9, and PR-10 exhibited alterations in their growth. These results are in agreement with several other reports that also showed alterations in the growth of the phage-resistant mutants (namely, a reduction in the growth rate) [62,63,64].

As the increase in the MOI from 1 to 1000 for phage AH-1 did not significantly increase the efficiency of treatment, the next experiments were performed with a MOI of 1. Although bacterial reduction with phage AH-1 occurs sooner at MOIs of 100 and 1000 (with a decrease of 2.8 and 3.0 log CFU/mL after 2 h of incubation (Figure 6A)), the initial doses of the phage AH-1 was not essential due to the phage self-perpetuating nature, revealed by a high increase of phage titers along with bacteria at MOI 1. The number of phage particles during the 12 h of incubation in the presence of the host at a MOI of 1 increased more (by 3.8 log PFU/mL) than at an MOI of 1000 (i.e., phage concentration remained similar to that of phage control) (Figure 6B). A high MOI may sometimes be a disadvantage for the success of phage treatment since the bacteria may be inactivated before replicating the phages. This can occur when an overload of phages simultaneously infects a bacterium, leading to lysis due to the presence of high concentrations of lysins, a phenomenon known as “lysis from without” [53,65,66]. The regrowth of the bacterial population which takes place after almost 6–10 h, even though it is very slow, could be considered as a constraint to phage therapy application. The increase in the MOI from 1 to 10, 100, or 1000 slightly increased the emergence of phage-resistant mutants, especially after 8 h of treatment. Similar results have already been observed in other studies [33,40,67,68]. Le et al. (2018), observed that the higher the MOI value, the sooner phage-resistant bacterial cells appeared [40]. A similar result was noted by Kim et al. (2012) for the phage PAS 1 against an *A. salmonicida*, indicating that bacterial resistance appeared after 3, 6, and 24 h at MOIs 10, 1, and 0.1, respectively [68].

One of the current challenges faced when performing phage biocontrol studies is to demonstrate its feasibility in vivo [27,28,69,70]. Hence, the efficiency of phage AH-1 was tested using artificially contaminated bivalves. The results of the in vivo experiments showed that phage AH-1 inactivated *A. hydrophila*, but that the efficacy was lower than that observed in vitro (with a maximum inactivation of 1.0 log CFU/g and 7.7 log CFU/mL, respectively) when compared to the non-treated samples. Similar results have been reported in other studies [27,28]. Pereira and colleagues observed that the efficiency of the single suspensions of phages phT4A and ECA2 and the phage cocktail (phT4/ECA2) used to treat cockles artificially contaminated with *E. coli* (with maximum inactivation of 2.0 log CFU/g) was lower than that obtained in in vitro assays (with maximum inactivation of 5.8, 4.7 and 6.2 log CFU/mL, respectively) [28,59]. In another study, the *S. typhimurium* inactivation rate in vitro using the phage cocktail phSE-2/phSE-5 (with maximum reduction of 2.0 log CFU/mL) was higher than the results recorded in vivo (with maximum reduction of 0.7 log CFU/g) [27,44]. However, the natural bacterial concentration at the beginning of the experiments was considerably high (2.7 log CFU/mL) when compared with similar studies (0.8–1.7 log CFU/mL) [27,44]. This high concentration of natural bacterial species may have hindered the inactivation success if those species are not within the bacteriophage’s infection range. Only around 1.7 log CFU/mL of the initial bacteria present in the bivalves at the beginning of the experiments (4.5 log CFU/mL) were added to the bivalves, corresponding to *A. hydrophila.* Therefore, more than 44% of the added *A. hydrophila* cells were inactivated. Bivalves display an uneven and large (internal) surface area, which physically limits the distribution of phage particles and prevents them from reaching their bacterial targets, therby impairing phage replication. On the other hand, the bivalve immune system can remove some of the added phages, hindering the bacterial inactivation. The concentration of phage particles produced in the presence of *A. hydrophila* in the in vitro experiments (an increase of 3.8 log PFU/mL) was higher than that observed in the in vivo experiments (phage concentration in the presence of hosts remains constant).

The reduction of *A. hydrophila* concentration by phage AH-1 in both in vitro and in vivo experiments is an important step forward in the development of an efficient and ecofriendly complement to depuration. However, in order to transfer this technology to industry, more studies are needed using naturally contaminated bivalves, first on a laboratory scale and, afterwards, scaling up to industrial conditions. Further evaluation trials must still be performed (e.g., whole-genome sequencing) in order to identify the presence of genes encoding toxins and/ or antibiotic resistance.

## 4. Materials and Methods

The selection of appropriate phages to be used in phage therapy represents a critical step towards achieving the successful phage-mediated control of pathogenic bacteria. Phages were characterized for their morphology, host-range, efficiency of plating, growth parameters (latent period and burst size), and frequency of emergence of phage resistant mutants. To evaluate the potential of three new phages to control *A. hydrophila,* and to select the best phage-inactivation conditions to be used in the bivalves decontamination, in vitro assays were performed. The first in vitro experiments were performed in Tryptic Soy Broth medium (TSB; Liofilchem, Roseto degli Abruzz, Italy) using phages individually or combined in cocktails (two or all the three phages mixed together) at a MOI of 1. As the major concern related to the use of phages to control infections is the emergency of phage-resistant mutants, the frequency of emergence of phage resistant mutants was determined using single phage suspensions and phage cocktails. Before application of phages to inactivate *A. hydrophila* during bivalves depuration, it is important to characterize in vitro the dynamics of phage-host replication. The kinetic theory of phage therapy predicts that the MOI could be critical to bacterial inactivation efficiency. For this reason, in a second step, bacterial inactivation was determined using the best phage selected in experiments above at MOIs of 1, 10, 100, and 1000. Then, the best in vitro phage treatment conditions were used in the in vivo assays using artificially contaminated bivalves with *A. hydrophila*.

### 4.1. Bacterial Strains and Growth Conditions

The bacterial strains used in this study are listed in Table 1. The bacterial strain *A. hydrophila* (ATCC 7966) was used in this study as a phage host. *Staphylococcus aureus* (ATCC 6538)*,* *S. typhimurium* (ATCC 13311 and ATCC 14028), *E. coli* (ATCC25922 and 13706), *V. parahaemolyticus* (DSM 27657), *V. anguillarum* (DSM 21597), *A. fischeri* ATCC 49387, *P. damselae damselae* (DSM 7482), *S. flexneri* (DSM 4782), *L. monocytogenes* (NCTC1194), *L. innocua* (NCTC 11288), and *A. salmonicida* (CECT 894) were purchased from the ATCC, DSM, NCTC, and CECT collections, respectively. Bioluminescent *E. coli* was used in previous works [71]. *A. caviae* CECT 838, *A. hydrophila* 839 and *Aeromonas* IR13 were isolated using water from the Vouga river [72]. Fresh plate bacterial cultures were kept in solid Tryptic Soy Agar medium (TSA; Liofilchem, Roseto degli Abruzzi, Italy) at 4 °C. Prior to each essay, one isolated colony was aseptically transferred to 10 mL of TSB (Liofilchem, Roseto degli Abruzzi, Italy) and was grown overnight at 25 °C or 37 °C. An aliquot of this culture (100 µL) was aseptically transferred to 10 mL of fresh TSB medium and grown overnight at 25 °C or 37 °C to hit an optical density (O.D. 600) of 0.8, corresponding to around 10^9^ cells for mL.

### 4.2. Phage Isolation and Purification

Phages AH-1, AH-4, and AH-5 were isolated from sewage water samples collected in the sewage network of Aveiro (SIMRIA Multi Sanitation System of Ria de Aveiro—station EEIS9) gathered at different times. One hundred millilitres of water was filtered through 0.45 µm pore size polycarbonate membranes (Millipore, Bedford, MA, USA). Filtered water was added to 100 mL of a twice concentrated TSB medium with 1 mL of a fresh culture of the host, *A. hydrophila* (ATCC 7966). The mixtures were incubated at 25 °C for 18 h at 80 rpm, and afterwards centrifuged at 10,000× *g* for 10 min at 4 °C and filtered through a polyethersulphate layer with a 0.22 µm pore size (Merck-Millipore, Darmstadt, Germany). Suspensions were stored at 4 °C and the titer was determined by the double-layer agar method [73]. Successive dilutions of the suspensions were done in phosphate-cradled saline (PBS) [137 mmol^−1^ NaCl (Sigma, St. Louis, MO, USA), 8.1 mmol^−1^ Na_2_HPO_4_·2H_2_O (Sigma, St. Louis MO, USA), 2.7 mmol^−1^ KCl (Sigma, St. Louis, MO, USA), and 1.76 mmol^−1^ KH_2_PO_4_ (Sigma, St. Louis, MO, USA), pH 7.4). 500 µL of each dilution, along with 200 µL of fresh bacterial culture, were mixed in 5 mL of TSB 0.6% top agar layer [30 g/L TSB (Liofilchem, Roseto degli Abruzzi, Italy), 6 g/L agar (Liofilchem, Roseto degli Abruzzi, Italy), 0.12 g/L MgSO_4_ (Sigma, St. Louis, MO, USA), and 0.05 g/L CaCl_2_ (Sigma, St. Louis, MO, USA), pH 7.4] and poured over a TSA plate. Plates were incubated at 25 °C and observed for the presence of lytic plaques after 12 h. One single plaque was selected from the agar and added to TSB medium with a fresh culture of the host. The sample was centrifuged, being the supernatant used as a phage source for a second isolation procedure. Three successive single-plaque isolation cycles were performed in order to acquire pure phage stocks. All lysates were centrifuged at 10,000× *g* for 10 min at 4 °C, to remove bacteria or bacterial debris. The phage suspensions were kept at 4 °C.

Phage stocks were prepared using *A. hydrophila* as the host. Phage stocks were prepared from the phage suspensions purified in SM buffer (0.1 M NaCl (Sigma-Aldrich, St. Louis, MO, USA), 20 mM Tris-HCl (Sigma, St. Louis, MO, USA), and 8 mM MgSO_4_ (Sigma, St. Louis, MO, USA), pH 7.5). After incubation, the stock culture of *A. hydrophila* in the exponential growth phase was centrifuged at 10,000× *g* for 10 min and the pellet was resuspended in 30 mL of SM buffer. Then, three hundred microliters of the phage stock were added to 30 mL of SM buffer with bacteria. The phage stocks were incubated at 25 °C under an orbital shaking set at 50 rpm. The lysate was centrifuged at 10,000× *g* for 10 min at 4 °C and the supernatant was filtered through a polyethersulphate membrane with a 0.22 µm pore size (Merck-Millipore, Darmstadt, Germany). The phage suspension was stored at 4 °C until and the titer was determined via the double-layer agar method [73] as described above. The plates were incubated at 25 °C for 12 h and the number of lysis plaques was counted. The results were expressed as plaque-forming units per millilitre (PFU/mL).

### 4.3. Electron Microscope Examination

Phage particles from high titter stock suspensions (10^10^ PFU/mL) were negatively stained with 2% uranyl acetate (Electron Microscopy Sciences, Hatfield, UK) and subjected to electron micrographs using a JEOL 1011 transmission electron microscope (JEDL USA Inc., Peabody, MA, USA) operating at 100 kV. The images were obtained with a Gatan CCD–Erlangshen ES100W.

### 4.4. Phage Host Range and Efficiency of Plating (EOP) Analysis

Spot testing according to the procedure described by [73], was done in order to assess the phage‘s host range, using the bacterial strains listed in Table 1. Five millilitres of TSB 0.6% agar, inoculated with 300 µL of fresh bacterial culture, was overlaid on solid TSA and spotted with 10 µL of the phage suspension. The plates were incubated at 25 °C and observed for the presence of lysis plaques after 12 h. A clear lysis zone at the spot determined bacterial sensitivity to the phage. Bacteria were differentiated according to either a clear lysis zone (+) or no lysis zone (−) (Table 1), depending on the clarity of the spot. Bacteria with positive spot tests (occurrence of clear lysis zone) were subjected to EOP using the double-layer agar method [73], as described before. The plates were incubated at 25 °C and observed for the presence of plaques after 12 h. The EOP for each bacterial host was calculated by comparison with the efficacy of *A. hydrophila* (host) (Table 1) and was calculated as (average PFU on target bacteria/average PFU on host bacteria) × 100 [74]. The EOP value obtained with the host strain was considered as EOP = 100%, and all values are presented in the manuscript as the mean of three independent measurements followed by their standard deviation.

### 4.5. One Step Growth Assays

Phage AH-1, AH-4, or AH-5 suspension (with a final concentration of 10^6^ PFU/mL) was added to 10 mL of a fresh bacterial culture of *A. hydrophila* (with a final concentration of 10^9^ CFU/mL) to have an MOI of 0.001 and the resulting suspension was incubated without shaking for 5 min at 25 °C [51]. The suspension was then centrifuged at 10,000× *g* for 5 min, the supernatant was discarded, and the pellet was resuspended in 10 mL of TSB and incubated at 25 °C. Samples of 1 mL were collected at time 0 and every 10 min up to 150 min of incubation and then immediately tittered by the double-layer agar method [73]. The plates were incubated at 25 °C and observed for plaques after 18 h. Three independent assays were performed.

### 4.6. Bacterial Kill Curves Using Single-Phage Suspensions and Phage Cocktails

Bacterial inactivation was determined using single phage suspensions (AH-1, AH-4, and AH-5) and phage cocktails (AH-1/AH-4, AH-1/AH-5, AH-4/AH-5, and AH-1/AH-4/AH-5; two or all phages were mixed with each phage at the same concentration) using the bacterium *A. hydrophila* at an MOI of 1. To obtain an MOI of 1, the exponential cultures of bacteria (final concentration of 10^5^ CFU/mL) and phage suspension (final concentration of 10^5^ PFU/mL) were inoculated in sterilized glass erlenmeyers with 30 mL of TSB medium and incubated at 25 °C without agitation (B + P). The phage titre was determined in triplicate by the double-layer agar method [73] after an incubation period of 12 h at 25 °C. Bacterial concentration was determined in triplicate in solid TSA medium through the drop-plate method after an incubation period of 24 h at 25 °C. Three independent experiments were performed for each condition.

### 4.7. Rate of Emergence of Bacterial Mutants Resistant to Phages

The development of *A. hydrophila* mutants, resistant to phages AH-1, AH-4, and AH-5, and phage cocktails AH-1/AH-4, AH-1/AH-5, AH-4/AH-5, and AH-1/AH-4/AH-5 was evaluated according to the procedure described by Filippov et al. (2011) [57]. In order to determine the frequency of phage-resistant bacteria, ten isolated colonies from a plate with sensitive bacteria were selected and inoculated into ten tubes with 5 mL of TSB medium and grown at 25 °C for 24 h (concentration around 10^9^ CFU/mL). Aliquots of 100 µL from the 10^0^ to 10^−2^ dilutions of the bacterial culture and aliquots of 100 µL of the phage from a stock solution of 10^9^ PFU/mL were inoculated into tubes containing TSB 0.6% agar, plated on TSA plates, and incubated at 25 °C for 3–5 days (since some of the phage-resistant mutants may grow slowly). Simultaneously, 100 µL aliquots of 10^−5^ to 10^−7^ dilutions of the bacterial culture were plated by incorporation on TSA plates without phage and incubated at 25 °C for 24 h. The calculation of the frequency of *A. hydrophila* spontaneous phage-resistant mutants was done by dividing the number of resistant bacteria (obtained from the bacteria grown in the presence of phage particles) by the total number of sensitive bacteria (prepared from the culture without phages) [57]. Three independent assays were performed.

Sensitive and phage AH-1-resistant colonies (phage AH-1 was selected for this experiment because it was the most efficient phage to control *A. hydrophila*) were picked up and purified by successive sub-culturing in TSA to remove attached phage particles and were used in further experiments (see Section 4.8).

A spot test on double agar plates was used to confirm the phage AH-1 resistance to phage-resistant strains and phage susceptibility of natural variant clones. To evaluate phages AH-4 and AH-5 sensitivity to phage AH-1-resistant strains, the spot test procedure was used. To confirm that the resistance of the bacterial isolates was stable through generations, the strains were passed through 10 consecutive subcultures in a liquid medium. Samples were taken from each subculture to assess bacterial resistance against phage infection using the double agar method.

### 4.8. Growth Curve of Phage-Resistant A. hydrophila Strains

To evaluate the growth of the ten AH-1-resistant bacteria and the ten natural variants, these and their parent strains were cultured in parallel on a 96 well plate with TSB medium at 25 °C. This phage was selected for these experiments because was the most efficient phage to control *A. hydrophila*. The O.D.600 nm of the cultures was measured at 0, 2, 4, 6, 8, 10, 12, and 24 h using a microplate photometer (Multiskan FC, Thermo Fischer). Three independent experiments were performed for each condition.

### 4.9. Bacterial Kill Curves at Different MOIs

*A. hydrophila* (with a final concentration of 10^5^ CFU/mL) inactivation by the phage AH-1 (final concentrations of 10^5^, 10^6^, 10^7^ and 10^8^ PFU/mL) was evaluated at MOI 1, 10, 100 and MOI 1000.

For each assay, two controls were included: the bacterial control (BC) and the phage control (PC). The bacterial controls were only inoculated with *A. hydrophila* and the phage controls were only inoculated with the phage suspensions. Controls and test samples (BP, bacteria plus phage) were incubated exactly in the same conditions and aliquots were collected at time 0 and after 2, 4, 6, 8, 10, and 12 h of incubation. In all assays, the phage titre was determined in triplicate by the double-layer agar method [73] after a 12 h incubation at 25 °C. Bacterial concentration was determined in triplicate in solid TSA medium through the drop-plate method after a 24 h incubation at 25 °C. Three independent assays were performed for each condition.

### 4.10. Phage Application during Cerastoderma edule Depuration in Static Seawater

#### 4.10.1. Collection of *C. edule* Samples

Cockles were selected as biological models to test the efficacy of phage biocontrol against *A. hydrophila* and were purchased from Mar de Sensações Lda. (Gafanha da Nazaré, Portugal), a bivalve wholesaler, after being depurated according to industrial processing protocols (48 h at 15–16 °C in seawater irradiated with UV–C).

Live cockles were transported to the laboratory in a container with seawater with controlled temperature (16 ± 1 °C), under an oxygen saturated atmosphere. Experiments were performed no more than 30 min post-collection.

#### 4.10.2. Depuration of Artificially Contaminated Cockles in the Presence of Phage AH-1 in a Static System

The efficacy of phage AH-1 (selected according to the results of the tests described above) was evaluated during depuration in a static system.

Cockles were kept in independent tanks (10 cm length × 9 cm width × 15 cm height), acting as a static system, filled with 0.6 L of sterile synthetic seawater [prepared by mixing a synthetic salt brand (Tropic Marin Pro Reef salt—TropicMarine, Wartenberg, Germany) with water purified by a reverse osmosis system (Aqua-win RO-6080, Kaohsiung, Taiwan) and then filtered through a 0.2 μm membrane (Millipore, Bedford, MA, USA)] and equipped with an aerator for 12 h before being infected with *A. hydrophila*. The temperature was maintained at 16 ± 1 °C, pH at 8.0 ± 0.2, salinity at 35, and dissolved oxygen above 5.5 mg/L during the experiment. After the adaptation period, cockles were washed with synthetic seawater and placed in independent tanks filled with synthetic seawater. The concentration of *Aeromonas* in cockles was determined before the decontamination procedure using the specific medium Glutamate Starch Phenol Red Agar (GSP, Liofilchem, Roseto degli Abruzzi, Italy). However, during the depuration of artificially contaminated cockles, the bacterial reduction was only evaluated by determining the concentration of cultivable bacteria. Three cockles were randomly selected from each tank, weighing about ten grams each. The flesh and intra-valvular liquid (FIL) of the cockles were pooled, blended in 90 mL of alkaline peptone water (Liofilchem, Roseto degli Abruzzi, Italy) and homogenized in a Bag Mixer 400 (Interscience, Saint Nom la Brétèche, France). The homogenized samples were then serially diluted ten-fold and 1mL from each dilution was spread on both GSP and TSA plates. All plates were incubated at 25 °C for 24 h.

A total of 4 groups of cockles were randomly formed, each group containing three replicates of 24 animals (24 specimens/tank × 4 groups × 3 replicates = 288 cockles). *A. hydrophila* was added to 2 of the 4 groups to obtain a final concentration of 10^5^ CFU/mL. In the other 2 groups, no *A. hydrophila* was added. Cockles from the 4 groups remained for 12 h in the tanks under the same conditions. Following 12 h of incubation, cockles were washed with sterilized artificial seawater and placed in clean tanks with no *A. hydrophila* contamination. From the 2 groups of cockles infected with *A. hydrophila*, 1 was inoculated with phage AH-1 (final concentration of 10^5^ PFU/mL) at an MOI of 1 (test tanks—BP), while the remaining group was not inoculated with any phage (bacteria control—BC). Concerning the 2 groups of cockles not infected with *A. hydrophila*, one of them was inoculated with phage AH-1 (phage control—PC) and the remaining group was not inoculated with the phage (cockle control—CC). All 4 groups were incubated exactly under the same conditions. Cockles of test tanks and controls were sampled at time 0 and 3, 6 and 12 h after phage addition. At each sampling time, three cockles were randomly selected from each tank, weighing about ten grams each. The flesh and intra-valvular liquid (FIL) of the cockles were pooled, blended in 90 mL of alkaline peptone water (Liofilchem, Roseto degli Abruzzi, Italy), and homogenized in a Bag Mixer 400 (Interscience, Saint Nom la Brétèche, France). The homogenized samples were then serially diluted ten-fold and 1mL from each dilution was spread on the non-specific TSA plates. All plates were incubated at 25 °C for 24 h. The counts in the TSA medium allowed the detection of the added *A. hydrophila*, as well as other cultivable bacteria that were already present in cockles. Phage titer was determined in duplicate using the double-agar layer technique [73] after an incubation period of 12 h at 25 °C. Three independent experiments were performed in different periods to secure independent replication.

### 4.11. Statistical Analysis

The statistical analysis of data was performed using the GraphPad Prism software 6.01, San Diego, California, USA. Normal distribution of the data was checked by a Kolmogorov–Smirnov test and the homogeneity of variance was assessed by Levene’s test. The significance of bacterial and viral concentrations between the treatments and during the experiments was tested using two-way ANOVA with repeated measures and Tukey’s multiple comparison post-hoc test (Section 2.5, Section 2.8 and Section 2.9). For different treatments, the significance of differences was evaluated by comparing the result obtained in the test samples with the results obtained for the correspondent control samples, for the different times. Two-way ANOVA was used to analyse the statistical differences between the growth curves of the sensitive and the phage-resistant bacteria during the sampling time (Section 2.7). A value of *p* < 0.05 was considered to be statistically significant.

## 5. Conclusions

In the present work, all phages efficiently inactivated the pathogenic bacterium *A. hydrophila* while inducing a low frequency of phage-resistant bacterial mutants during in vitro applications. Furthermore, phage AH-1 successfully improved the efficiency of depuration in a static water system. The results of this study provide further evidence that phages can safely inactivate pathogenic bacteria and may be successfully combined with industrial practices already used to improve their efficacy.

## Figures and Tables

**Table 1:** Host range of phages AH-1, AH-4, and AH-5 determined on 19 bacterial strains. Clear lysis zone, (+); no lysis zone, (−). The plating with the host strain was considered as EOP = 100%.

**Species**	**Infectivity of Phage**			**Efficacy of Plating (%)**		
	**AH-1**	**AH-4**	**AH-5**	**AH-1**	**AH-4**	**AH-5**
*Aeromonas hydrophila* ATCC 7966	+	+	+	100	100	100
*Aeromonas hydrophila* 839	+	+	+	83.3 ± 3.52	91.14 ± 5.73	76.19 ± 2.61
*Aeromonas caviae* 838	−	−	−	0	0	0
*Aeromonas salmonicida* CECT 894	−	−	−	0	0	0
*Aeromonas* IR13	−	−	−	0	0	0
Bioluminescent *Escherichia coli*	−	+	-	0	3.31 ± 0.50	0
*Escherichia coli* ATCC 25922	−	−	−	0	0	0
*Escherichia coli* ATCC 13706	−	−	−	0	0	0
*Listeria innocua* NCTC 11288	−	−	−	0	0	0
*Listeria monocytogenes* NCTC 1194	−	−	−	0	0	0
*Photobacterium damselae damselae* DSM 7482	−	−	−	0	0	0
*Salmonella typhimurium* ATCC 13311	+	+	−	56.10 ± 2.32	10.17 ± 0.35	0
*Salmonella typhimurium* ATCC 14028	−	−	−	0	0	0
*Salmonella typhimurium* WG49	−	−	−	0	0	0
*Shigella flexneri* DSM 4782	−	−	−	0	0	0
*Staphylococcus aureus* ATCC 6538	−	−	−	0	0	0
*Vibrio parahaemolyticus* DSM 27657	−	−	−	0	0	0
*Vibrio anguillarum* DSM 21597	−	−	−	0	0	0
*Aliivibrio fischeri* ATCC 49387	−	−	−	0	0	0

**Table 2:** Frequency of emergence of *A. hydrophila* spontaneous phage-resistant.

**Phages/Phage Cocktails**	**Control Sample (CFU/mL)**	**Sample Treated with Phages**	**Frequency of Mutants**
AH-1	1.24 ± 0.45 × 10^9^	3.85 ± 2.77 × 10^4^	3.10 × 10^−^^3^
AH-4	2.41 ± 0.93 × 10^9^	2.75 ± 1.20 × 10^4^	1.14 × 10^−^^3^
AH-5	3.27 ± 0.11 × 10^9^	1.64 ± 0.35 × 10^4^	5.02 × 10^−^^4^
AH-1/AH-4	6.43 ± 0.12 × 10^9^	5.32 ± 2.46 × 10^4^	8.26 × 10^−^^4^
AH-1/AH-5	8.15 ± 0.36 × 10^9^	5.22 ± 3.12 × 10^4^	6.40 × 10^−^^4^
AH-4/AH-5	2.17 ± 0.58 × 10^9^	1.55 ± 1.15 × 10^4^	7.13 × 10^−^^4^
AH-1/AH-4/AH-5	9.23 ± 2.08 × 10^9^	5.53 ± 3.24 × 10^4^	5.99 × 10^−^^4^

## Data Availability

Data is contained within the article.
